# The Activities of Lysyl Hydroxylase 3 (LH3) Regulate the Amount and Oligomerization Status of Adiponectin

**DOI:** 10.1371/journal.pone.0050045

**Published:** 2012-11-29

**Authors:** Heli Ruotsalainen, Maija Risteli, Chunguang Wang, Yu Wang, Marjo Karppinen, Ulrich Bergmann, Ari-Pekka Kvist, Helmut Pospiech, Karl-Heinz Herzig, Raili Myllylä

**Affiliations:** 1 Department of Biochemistry, University of Oulu, Oulu, Finland; 2 Department of Pharmacology and Pharmacy, Faculty of Medicine Complexes, University of Hong Kong, Pokfulam, Hong Kong, China; 3 Institute of Biomedicine, Department of Physiology, and Biocenter Oulu, University of Oulu, Oulu, Finland; 4 Department of Psychiatry, Kuopio University Hospital, Kuopio, Finland; Graduate School of Medicine, the University of Tokyo, Japan

## Abstract

Lysyl hydroxylase 3 (LH3) has lysyl hydroxylase, galactosyltransferase, and glucosyltransferase activities, which are sequentially required for the formation of glucosylgalactosyl hydroxylysines in collagens. Here we demonstrate for the first time that LH3 also modifies the lysine residues in the collagenous domain of adiponectin, which has important roles in glucose and lipid metabolism and inflammation. Hydroxylation and, especially, glycosylation of the lysine residues of adiponectin have been shown to be essential for the formation of the more active high molecular weight adiponectin oligomers and thus for its function. In cells that totally lack LH3 enzyme, the galactosylhydroxylysine residues of adiponectin were not glucosylated to glucosylgalactosylhydroxylysine residues and the formation of high and middle molecular weight adiponectin oligomers was impaired. Circulating adiponectin levels in mutant mice lacking the lysyl hydroxylase activity of LH3 were significantly reduced, which indicates that LH3 is required for complete modification of lysine residues in adiponectin and the loss of some of the glycosylated hydroxylysine residues severely affects the secretion of adiponectin. LH mutant mice with reduced adiponectin level showed a high fat diet-induced increase in glucose, triglyceride, and LDL-cholesterol levels, hallmarks of the metabolic syndrome in humans. Our results reveal the first indication that LH3 is an important regulator of adiponectin biosynthesis, secretion and activity and thus might be a potential candidate for therapeutic applications in diseases associated with obesity and insulin resistance.

## Introduction

Lysyl hydroxylase 3 (LH3) is a multifunctional enzyme possessing lysyl hydroxylase (E.C. 1.14.11.4), collagen galactosyltransferase (E.C. 2.4.1.50) and glucosyltransferase (E.C. 2.4.1.66) activities [1], [2], which catalyzes the formation of specific glucosylgalactosylhydroxylysine (Glc-Gal-Hyl) residues in the Y position of X-Y-Gly triplets of collagens [3]. The modifications of lysine residues in collagens take place in the lumen of the endoplasmic reticulum (ER) [4]. In addition to its ER localization, LH3 resides in the extracellular space of tissues and in serum [5], [6]. The extracellular function of LH3 is still not fully understood, although, the presence of glycosyltransferase activities of LH3 in the extracellular space has been shown to be important for cell growth and viability [7].

The absence of LH3 in developing homozygous LH3 (LH3^−/−^) knockout mouse embryos leads to death around E9.5. The secretion of type IV collagen is blocked in these embryos that lack LH3 catalyzed Glc-Gal-Hyl residues, and this disrupts formation of the basement membranes that support tissues e.g. blood vessels [8]. The absence of Glc-Gal-Hyl residues also prevents the oligomerization of type VI collagen, and leads to impaired secretion of type VI collagen tetramers in LH3^−/−^ knockout mouse embryonic fibroblasts (MEFs). Overall, the lack of LH3 prevents the formation of hydroxylysine linked Glc-Gal structures in all collagen types studied. [9].

The inactivation of lysyl hydroxylase activity of LH3 by a Asp669Ala point mutation (LH mutant mice), however, does not disturb embryonic development, although the slight underglycosylation of hydroxylysine residues in type IV and VI collagens causes their abnormal distribution in the extracellular matrix in LH mutant cells. Particularly the underglycosylated type VI collagen forms massive aggregates in tissues of LH mutant mice [9]. In humans, an LH3 protein with reduced lysyl hydroxylase, galactosyltransferase and glucosyltransferase activities is associated with a severe, unique connective tissue disorder [10]. Recently it was shown that even a moderate decrease in the amount of LH3 in human and in heterozygous LH3 knockout (LH3^+/−^) mice is enough to cause abnormalities in the deposition and organization of the extracellular matrix [11].

There are many proteins that are not members of the collagen family, but nevertheless have a short collagenous domain in their structure. Glc-Gal-Hyl residues are also present in the collagenous domain of these proteins, but little is known about their biological effects on the structure and function of the proteins. Adiponectin (also known as ACRP30 and AdipoQ), the major insulin-sensitizing hormone secreted by adipose tissue, contains a short collagenous domain of 22 X-Y-Gly triplets. Decreased plasma concentrations of adiponectin have been reported to have a causal role in the development of insulin resistance, type 2 diabetes and metabolic syndrome [12]–[15]. In obesity, adipokines are generally increased, but adiponectin is down-regulated by an unknown mechanism.

Adiponectin is found in circulation as three oligomers: Low Molecular Weight (LMW, trimer), Middle Molecular Weight (MMW, hexamer) and High Molecular Weight (HMW) [16]. Four conserved Glc-Gal-Hyl residues in the collagenous domain of adiponectin contribute to the insulin-sensitizing activity of the protein [17] and have a significant role in the assembly of oligomers, especially for the formation of HMW adiponectin [18]–[20]. Several observations suggest that the HMW is the most active form of adiponectin, and decreased serum levels of the HMW form correlate with metabolic syndrome traits [16], [21].

Recent studies suggest that the regulation of the posttranslational lysine modifications in the collagenous domain is the key event in determining the function of adiponectin [16]. In this study we investigated the possibility that LH3 is responsible for lysine modifications in adiponectin and whether changes in LH3 activities regulate the oligomerization and functions of adiponectin. Our results indicate that LH3 activities are required for the posttranslational lysine modifications of adiponectin.

## Materials and Methods

### Ethical statement

All mouse experiments were approved by the National Animal Ethics Committee of Finland (Permit number: ESLH-2007-10387/Ym-23). Blood samples were collected by orbital bleeding under medetomin-ketamin terminal anesthesia.

### Mouse and cell lines

The generation of LH3 knockout and LH mutant mouse lines has been described previously [8]. In LH mutant mice the lysyl hydroxylase activity of LH3 is specifically inactivated by a knock-in point mutation, Asp669Ala. The mice have been backcrossed to the C57BL/6 strain for 6 to 9 generations and were maintained under 12-hour light and dark cycles with ad libitum access to water and a standard chow diet. For diet studies, four month old LH mutant and wild type female mice weighing on average 22.7±1.3 g and 22.5±1.9 g, respectively, were fed a high fat diet (60% kcal from fat, D12492, Research diets) for four months. We chose female mice for the study because it was reported that females have approximately 50–60% of adiponectin in the HMW form in serum, whereas in males only 25–40% is in HMW form [21], [22].

LH3^−/−^ knockout mouse embryonic fibroblasts (MEFs) and LH mutant MEFs were derived as previously described [9]. All cell lines were cultured in Dulbecco's Modified Eagle Medium (DMEM) (Invitrogen) supplemented with 10% fetal calf serum (Promocell), penicillin/streptomycin, and 50 µg/ml ascorbic acid at 37°C in a humidified atmosphere of 95% air and 5% CO_2_.

### Production of recombinant adiponectin and LH3

Mammalian expression vectors pcQF encoding a FLAG tagged mouse adiponectin [17] and pcDNA3 containing human LH3 with a signal peptide and a c-Myc-tag at the amino-terminus [23] were transfected into LH3^−/−^ knockout, LH mutant and wild type MEFs using a MEF Nucleofector® Kit and Amaxa® Nucleofector® technology (Lonza). The following day the culture medium was changed to serum free DMEM, penicillin/streptomycin, and 50 µg/ml ascorbic acid, and the cells were cultured for 48 h. The medium was collected and recombinant adiponectin was affinity purified using anti-FLAG M2 affinity gel (Sigma), eluted with 2× SDS loading buffer or with the FLAG peptipe (Sigma) and analyzed on SDS-PAGE and immunoblot, or the medium was concentrated by Amicon Ultra centrifugal filters (10 kDa MWCO, Millipore) and applied to a gel filtration column.

### Mass spectrometry analysis

Purified recombinant FLAG tagged mouse adiponectin was fractionated on SDS-PAGE, and the band that corresponded to adiponectin on the Coomassie blue-stained gel was excised. The gel pieces were destained with three 5 min wash cycles using destaining buffer (50 mM ammoniumbicarbonate in 40% (v/v) acetonitrile/water), followed by incubation for 30 min at room temperature with 20 mM DTT in the same buffer, and subsequent addition of iodoacetamide to 40 mM. After incubation for 30 min at room temperature, the gel pieces were washed once with destaining buffer and twice with water, before dehydration with acetonitrile and drying in a speed vac device. The dried gel pieces were rehydrated with trypsin solution (5 ng/µl in 25 mM ammoniumbicarbonate 10/90% (v/v) acetonitrile water) stored on ice for about 30 min and then incubated over night at 37°C. Tryptic peptides were extracted from the gel with 20 µl 30% (v/v) acetonitrile/water by ultrsonication for 10 min. Peptide mass fingerprints were measured on an UltrafleXtreme MALDI Tof mass spectrometer (Bruker Daltonics) in reflector mode. To that end, 0.5 µl, of the peptides solution was spotted on an anchor chip samples stage, dried, and overlaid with α-cyanohydroxicynnamic acid as matrix, following the protocols of the manufacturer. The instrument was tuned for peptide measurements between m/z 700 and 5000, external calibration was achieved with standard peptides including insulin to cover the higher mass range. Protein identification and data analysis was done with the Flex Analysis and Biotools software packages (Bruker Daltonics) using Mascot (Matrix Science) as search engine.

### Enzyme-linked immunosorbent assay and gel filtration chromatography

The total adiponectin level was measured using either an in-house ELISA [24] or commercial ELISA kit (R&D Systems) specific for mouse adiponectin. Leptin level was measured using Mouse leptin ELISA kit (Millipore).

For the analysis of the different oligomers of adiponectin cell culture medium or mouse serum was fractionated by an Äkta purifier chromatography system using a Superdex 200 10/300 GL gel filtration column and eluted with 150 mM NaCl, 50 mM phosphate buffer, pH 7.2 or with 25 mM Hepes, 150 mM NaCl, 1 mM CaCl_2_, pH 7.4, at a flow rate of 0.5 ml/min. Fractions of 0.215 ml were collected and the concentration of adiponectin was determined by ELISA as above, or by immunoblot analysis. Corresponding gel filtration fractions of separate serum samples were pooled before ELISA analysis.

### Immunoblot analysis

In order to analyze the amount of adiponectin, equal volumes of cell culture medium or diluted serum were loaded onto a 15% SDS-PAGE. For the analysis of the molecular weight of adiponectin, 5 µl and 10 µl of diluted (1/300) wild type and LH mutant serum samples, respectively, and equal volumes of cell culture medium were separated near the end of gel. For immunoblot analysis adipose tissue was homogenized with 0.1 M glycine, 0.02 M Tris-HCl pH 7.8, 1% Igepal, or 25 mM Hepes pH 7.5, 5 mM EDTA, 5 mM EGTA, 100 mM NaCl, 10% glycerol, 1% Triton X-100 buffer including Complete EDTA-free protease inhibitor cocktail (Roche), and disrupted by brief sonication. The cell debris was removed by centrifugation. The protein concentrations were measured with Protein assay (Bio-Rad), and equal amounts of soluble protein were loaded onto the gel. The proteins were separated under reducing conditions by SDS-PAGE and transferred to an Immobilon-P transfer membrane (Millipore). For analysis of adiponectin oligomers equal volumes of cell culture medium were separated in non-reducing and non-heat-denaturing conditions [20]. The membranes were blocked with 5% milk powder in Tris buffered saline – Tween 20 and incubated with rabbit anti-adiponectin (Affinity BioReagents) followed by a horseradish peroxidase-conjugated anti-rabbit IgG (P.A.R.I.S. Biotech). Immunocomplexes were visualized using an ECL+ detection system (GE Healthcare) and Kodak XAR films or Molecular imager ChemiDoc XRS+ (Bio-Rad). The mobility of the adiponectin monomer was measured using CorelDraw software and size of the monomer was calculated using molecular weight standard curve. Quantification of adiponectin levels was performed with ImageQuant TL 7.0 (GE Healthcare) or Image Lab software (Bio-Rad).

### Measurement of blood parameters

Fasting (6 hours) blood samples were collected from anesthetized mice by orbital bleeding into Micro tubes with Z-gel (Sarsted), and serum was separated by centrifugation after 30 min. Serum total HDL and LDL cholesterol, triglycerides, FFA and lipase were analyzed by the Laboratory services of Oulu University Hospital, Finland. Serum glucose was measured during blood collection using an OneTouch® Ultra®2 glucometer and insulin levels with the Insulin (mouse) ELISA kit (Alpco Diagnostics).

### Quantitative real-time PCR

For gene expression analysis, RNA was isolated from epididymal adipose, muscle and liver tissue using Trizol reagent combined with a PureLink RNA mini kit (Invitrogen). The cDNA was synthesized from 0.5 µg of isolated total RNA using a Cloned AMV first-strand cDNA synthesis kit (Invitrogen). Quantitative Real-Time (RT) PCR was performed using a TaqMan® Universal PCR Master Mix (Applied Biosystems) and an ABI 7500 PCR System (Applied Biosystems). TaqMan® gene expression assay Mm00456425_m1 was used to amplify adiponectin. Primers and 5′ FAM-labeled probes for carnitine palmitoyltransferase (CPT-1), very long chain acyl-CoA dehydrogenase (VLCAD), acetyl-CoA carboxylase (Acaca) and phosphoenoylpyruvate carboxykinase (PEPCK-C) were designed as described elsewhere [25]. Quantification is based on actual efficiencies that were determined from standard curves run in parallel with the quantification samples. The results were normalized with GAPDH and β-actin (Pre-Developed TaqMan® assay reagents 4333760F and 4352933E, respectively, Applied Biosystems) as endogenous control for each sample. All measurements were performed in triplicate with 10 biological replicates. Expression was analyzed individually from RNA isolated from each of 10 mice for both genotypes. Average expression and confidence intervals were then determined for each genotype from the individual expression data.

### Statistics

The normality of the data set was analyzed with a D'Agostino-Pearson test and equality of the variances was tested with an F-test after which statistical analyses were performed using the Student's t-test with corresponding parameters.

## Results

### Multifunctional LH3 is needed for the hydroxylation and glycosylation of lysine residues in adiponectin

**Figure 1:**
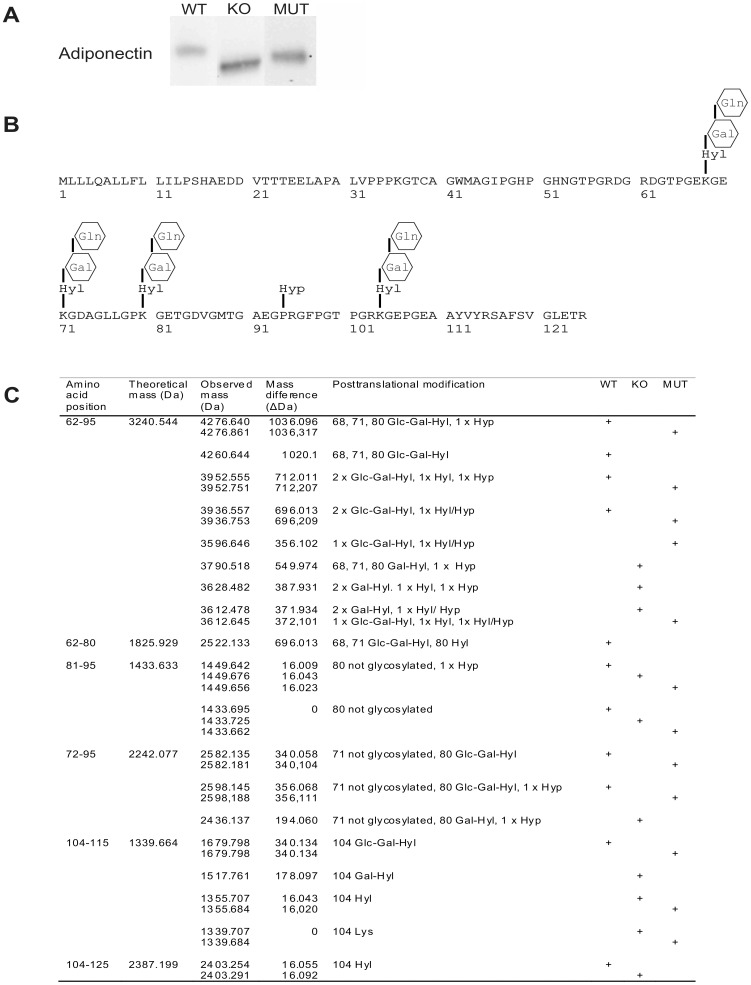
Immunoblot and mass analyses of adiponectin produced in LH3 manipulated cell lines. (A) Recombinant adiponectin was produced in MEF cells and the medium containing the secreted adiponectin was collected for the analysis. The immunoblot analysis of recombinant adiponectin monomers from the concentrated medium of wild type, LH3^−/−^ knockout and LH mutant MEF cells indicates a clear size difference. (B) Schematic picture of the collagenous domain of mouse adiponectin shows the positions of posttranslational modifications of lysine and proline residues reported earlier. (C) Mass spectrometry identification of peptides and modifications from tryptic digests of mouse adiponectin produced in wild type, LH3^−/−^ knockout and LH mutant MEFs. Abbreviations: WT = wild type; KO = LH3^−/−^ knockout; MUT = LH mutant; Hyl = hydroxylysine; Gal = galactosyl; Glc = glucosyl.

Our recent data show that LH3 catalyzes the formation of Glc-Gal-Hyl residues in collagens [9]. Similar posttranslational lysine modifications are also present in a number of other proteins with a collagenous domain. One of these proteins is adiponectin, in which the hydroxylated and glycosylated lysines contribute to the structure and function of the molecule [17]–[19]. In order to study whether LH3 also catalyzes the lysine modifications of adiponectin, our LH3^−/−^ knockout, LH mutant and wild type MEF cells were transiently transfected to produce recombinant mouse adiponectin [17]. The LH3^−/−^ knockout MEFs totally lack the LH3 protein, while in LH mutant MEFs only the lysyl hydroxylase activity of LH3 has been eliminated by substituting aspartate-669 with alanine [9]. Immunoblot analysis showed that the adiponectin monomers produced in LH3^−/−^ knockout (Fig. 1A middle) and LH mutant (Fig. 1A right) MEFs migrated faster than the wild type (Fig. 1A left) monomers, which suggests differences in the molecular weight and thereby in the posttranslational lysine modifications.

In order to confirm the immunoblot results tryptic peptide mixtures of recombinant adiponectin produced in LH3^−/−^ knockout, LH mutant and wild type MEFs were analyzed by mass spectrometry (Fig. 1B, 1C and S1). The theoretical molecular masses of the tryptic adiponectin peptides of collagenous domain are 1339.664 Da (amino acids 104–115) and 3240.544 Da (amino acids 62–95). The smaller of them can contain one Glc-Gal-Hyl residue on a lysine residue at position 104 causing a theoretical mass shift to 1679.765 Da. The longer peptide can be modified at up to three sites (68, 71, and 80), resulting in a peptide of 4260.845 Da. In addition, the proline at position 94 can be hydroxylated to give a peptide mass of 4276.840 (Fig. 1B) [17]. In our mass spectrometric analyses (Fig. 1C and S1) of recombinant adiponectin produced in wild type MEFs we saw fragments with masses of 4276, 4260 and 1679 Da as expected. In these fragments lysines 68, 71, 80 and 104 were modified to Glc-Gal-Hyl and the peptide with the mass of 4276 presumably contained also hydroxyproline at position 94. In addition, comparison of the experimentally derived mass spectrum with the theoretical one revealed fragments, which cover amino acids 62–95 with masses of 3952 and 3936 Da suggesting that one of the three Glc-Gal-Hyl residues is missing in the peptide. Variation in lysine modifications was further supported by the presence of fragments corresponding to amino acids 62–80, 81–95 and 72–95 of adiponectin, which suggests that lysines 80 and 71 are not always glycosylated. Furthermore, the mass corresponding to an oxidized fragment containing residues 104–125 suggests that hydroxylysine 104 is not always glycosylated. Trypsin cleavage is known to occur at C-terminal peptide bond of arginine, lysine and hydroxylysine but not at Glc-Gal-Hyl residue [26]. However cleavage was not always quantitative since fragment 104–125 contains an internal miscleaved arginine, and fragment 62–95 was found with several molecular weights suggesting a missed cleavage at an internal hydroxylysine.

Analysis of adiponectin produced in LH3^−/−^ knockout MEFs revealed a set of fragments with unique masses, which were not present in wild type recombinant adiponectin (Fig. 1C and S1). Peptides with masses 1679 Da and 4260 Da were completely disappeared in LH3^−/−^ knockout. Mass differences between LH3^−/−^ knockout and wild type fragments corresponded to the loss of Glc units in Glc-Gal-Hyl residues in adiponectin produced in LH3^−/−^ knockout MEFs. This suggests that LH3 is essential for the glucosylation of Gal-Hyl residues of adiponectin, but galactosylation and hydroxylation can be compensated by other lysyl hydroxylases and galactosyltransferases. Interestingly, the fragment 104–115 was also detected with a mass of 1339 Da indicating that lysine 104 is not always hydroxylated in adiponectin produced in LH3^−/−^ knockout MEFs, whereas in wild type adiponectin lysine 104 appeared to be at least hydroxylated.

Adiponectin produced in LH mutant MEFs was mainly trypsinized to fragments with similar masses as found in adiponectin produced in wild type MEFs (Fig. 1C and S1). However, intensity of the major peaks was decreased in LH mutant and the fragment corresponding to amino acids 62–95 with masses of 3596 and 3612 Da suggest that there is more variation in the glycosylation of lysines 68, 71 and 80 than in wild type adiponectin. Furthermore the fragment 104–115 was present with a mass of 1339 Da similarly as in LH3^−/−^ knockout produced adiponectin suggesting that LH3 preferentially hydroxylates lysine 104. Our mass analyses confirm adiponectin immunoblot data and clearly indicate that all enzymatic activities of LH3 are needed for the complete hydroxylation and glycosylation of lysine residues in the collagenous domain of adiponectin.

### Recombinant adiponectin produced in LH3^−/−^ knockout MEF cells does not form HMW and MMW oligomers

**Figure 2:**
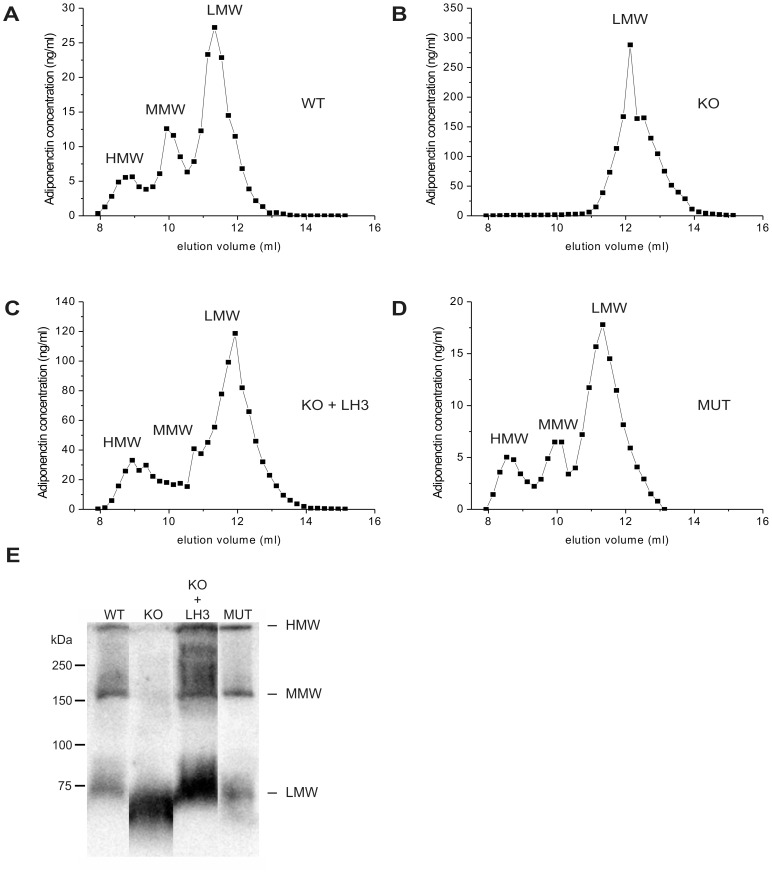
Oligomer distribution of recombinant adiponectin is altered in LH3^−/−^ knockout MEF cells. The oligomeric forms of recombinant adiponectin were separated with gel filtration chromatography and quantified by ELISA. Comparison of typical gel filtration elution profiles of recombinant adiponectin produced in (A) wild type and (B) LH3^−/−^ knockout MEFs indicates that HMW and MMW oligomers are not secreted from knockout cells. (C) Double transfection with adiponectin and LH3 constructs normalized the elution profile in LH3^−/−^ knockout MEFs. (D) Recombinant adiponectin produced in LH mutant MEFs formed similar oligomers as the wild type. The adiponectin oligomers corresponding to the peaks seen with gel filtration chromatography were also detected on immunoblot (E) separated under non-reducing and non-heat-denaturing conditions. Equal volumes of concentrated cell culture media were used in all analysis. Representative elution profiles are shown. Abbreviations: WT = wild type; KO = LH3^−/−^ knockout; LH3 = lysyl hydroxylase isoform 3; MUT = LH mutant.

Hydroxylation and glycosylation of the four conserved lysine residues in the collagenous domain of adiponectin is essential for its secretion and oligomerization into three oligomeric forms: LMW, MMW and HMW [16]. Our ELISA analysis of the distribution of recombinant adiponectin between transfected cells and the culture medium indicated inefficient secretion of adiponectin from the LH3^−/−^ knockout MEFs (Table 1). Wild type MEFs secreted 66% of the adiponectin produced into the medium, whereas LH3^−/−^ knockout MEFs secreted only 22%. In order to assess the maturation and secretion of recombinant adiponectin oligomers in the LH3^−/−^ knockout MEFs, the oligomer distribution was analyzed in the concentrated cell culture medium by gel filtration chromatography, ELISA and non-reducing and non-heat-denaturing immunoblot. Recombinant adiponectin was secreted from wild type MEFs as three oligomeric forms as expected with the distribution of 14% HMW, 21% MMW and 65% LMW of the total adiponectin (Fig. 2A) and these three major forms were also seen on immunoblot under non-reducing and non-heat-denaturing conditions (Fig. 2E). Recombinant adiponectin produced in the LH3^−/−^ knockout MEFs totally lacked the larger HMW and MMW forms, and only the trimeric LMW adiponectin was secreted to the cell culture medium (Fig. 2B and 2E). In addition, the LMW form synthesized in LH3^−/−^ knockout MEFs eluted later from a gel filtration column and migrated faster on immunoblots than wild type LMW (Fig. 2A, 2B and 2E) suggesting a lower molecular weight and thus less lysine glucosylation as seen in mass spectrometry. Transient double transfection of adiponectin and LH3 to LH3^−/−^ knockout MEFs restored the molecular weight of the recombinant adiponectin monomer to correspond to that of the adiponectin produced in wild type MEFs (not shown). In addition, the expression of adiponectin together with LH3 normalized the elution profile and oligomerization pattern in LH3^−/−^ knockout MEFs (Fig. 2C and 2E) to that seen in the wild type MEFs (Fig. 2A and 2E). Recombinant adiponectin produced in the LH mutant MEFs assembled to all adiponectin oligomers seen in wild type (Fig. 2D and 2E) and no obvious changes in elution and migration were detected. Our *in vitro* data clearly confirm the role of LH3 in the biosynthesis of adiponectin as a lysine modifying enzyme and thus reveals its importance for the secretion of high molecular weight oligomers into the extracellular space.

**Table 1:** Distribution of recombinant adiponectin in MEF cell lysate and cell culture media.

Cells	Cell medium		Cell lysate	
	ng/plate^A^	% of total^B^	ng/plate^A^	% of total^B^
MEF-wt	497	66.0±3.2	260	34.0±3.2
MEF-LH3^−/−^ knockout	839	22.2±1.3	2928	77.8±1.3

A Average of three experiments in which equal number of cells were used.

B The results are calculated as a percentage of adiponectin from the total adiponectin on a cell culture plate (ng in lysate+ng in media measured by ELISA) and presented as average values ± S.D. from 3 different transfections.

### Changes in the lysyl hydroxylase activity of LH3 affect the adiponectin level and modifications in mouse

Due to the lethality of the total LH3^−/−^ knockout [8] we used our heterozygous LH3 knockout (LH3^+/−^) and LH mutant (lysyl hydroxylase activity of LH3 inactivated by a point mutation Asp669Ala) mouse lines to further analyze the *in vivo* functions of LH3 on adiponectin biosynthesis. LH3^+/−^ mice are externally normal [8], but have ultrastructural changes in the skin [11]. In cell culture of LH3^+/−^ MEFs, a moderate decrease in LH3 activity due to loss of one *Plod3* allele affected the organization of the ECM [11]. The LH mutant mice showed defects in basement membranes and in collagen fibril organization due to underglycosylation of type IV and VI collagen [8], [9].

**Figure 3:**
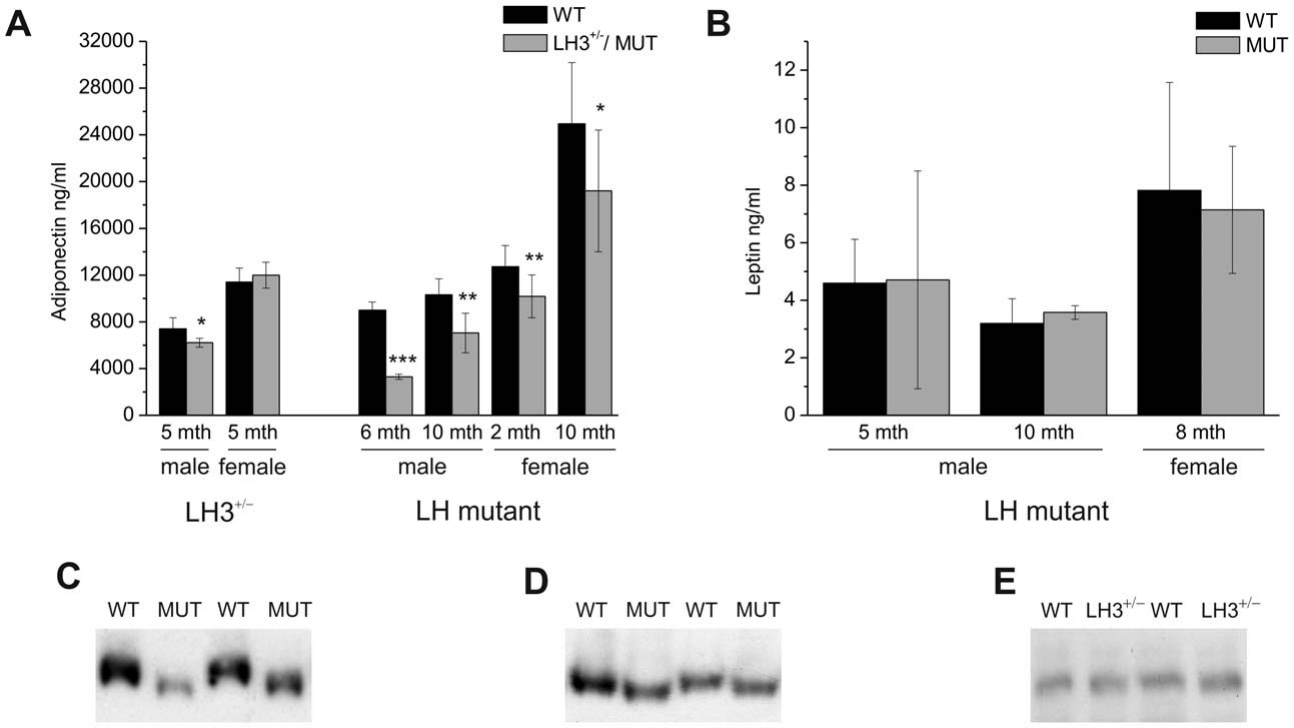
Total adiponectin levels in LH mutant mice and heterozygous LH3 knockout mice. (A) Total adiponectin levels were significantly reduced in the serum of LH mutant mice (MUT), but not in the heterozygous LH3 knockout (LH3^+/−^) mice. Adiponectin level analyses were done from the serum of the 5 months old male (n = 6 wt, 5 ko^+/−^) and female (n = 6 wt, 6 ko^+/−^) LH3^+/−^ mice, and 6 months (n = 6 wt, 5 mut) and 10 months (n = 7 wt, 5 mut) old male, and 2 months (n = 7 wt, 5 mut) and 10 months (n = 9 wt, 7 mut) old female LH mutant mice with ELISA. (B) Total leptin levels were comparable in LH mutant and wild type mice. Leptin analyses were done from the serum of the 5 months (n = 6 wt, 4 mut) and 10 months (n = 4 wt, 3 mut) old male, and 8 months (n = 5 wt, 5 mut) old female LH mutant mice with ELISA. (C) Adiponectin monomers from LH mutant and wild type serum exhibited differences in electrophoretic mobility. In order to evaluate the electrophoretic mobility of adiponectin, different volumes, 5 µl and 10 µl, of diluted (1/300) serum of wild type and LH mutant mice were separated near the end of a 15% SDS-PAGE. (D) The immunoblot analysis of adipose tissue homogenate (41 µg of protein) of wild type and LH mutant male mice did not show differences in the levels of adiponectin monomers, but the electrophoretic mobility of LH mutant adiponectin was clearly increased compared to that of the wild type. (E) In the adipose tissue of LH3^+/−^ mice (50 µg of protein loaded on the gel) no shift in the mobility or in the amount of adiponectin monomers was detected. In immunoblots C, D and E two representative wild type, LH mutant and LH3^+/−^ samples are shown. The values represent the average ± SD of the experiments. P values were calculated using unpaired homoscedastic student t-test with two-tailed distribution. * p<0.05, ** p<0.01, *** p<0.001.

In order to determine how the changes in the activities of LH3 affect the circulating level of adiponectin, we used ELISA to measure the total adiponectin level in sera of the LH3^+/−^ and LH mutant mice. In the 5 months old LH3^+/−^ female mice the concentration of adiponectin in serum was comparable with the wild type, whereas in male LH3^+/−^ mice a 16% decrease was measured (Fig. 3A). Comparable adiponectin levels in female LH3^+/−^ and wild type serum suggest that the level of LH3 protein produced from one functional allele of the LH3 gene, *Plod3*, is enough for the catalysis of lysine modifications in adiponectin, even though a slight decrease was detected in LH3^+/−^ male mice. In the LH mutant mice more obvious changes were detected in the serum levels of adiponectin. The circulating adiponectin level was significantly reduced both in male (37% and 68% of wild type, at ages 6 months and 10 months respectively) and female (80% and 77% of wild type, at ages 2 and 10 months respectively) LH mutant mice (Fig. 3A). In males the drop was more dramatic than in females as was seen previously in the LH3^+/−^ mice. In general, the adiponectin level was higher in females than in males, which supports earlier reports [27]–[29]. The leptin level was analyzed in order to determine whether other adipokines are also affected in LH mutant mice. The leptin level in the sera of LH mutant mice was comparable to wild type in 5 and 10 months old male and 8 months old female mice (Fig. 3B), which indicates that biosynthesis of adipokines without lysine modifications is not affected in LH mutant mice. The reduction in the adiponectin level in the serum of the LH mutant indicates that the loss of the lysyl hydroxylase activity of LH3 reduces the amount of circulating adiponectin.

The immunoblot analyses of serum samples of LH3^+/−^ knockout mice showed no obvious changes in the mobility or in the amount of adiponectin in agreement with the ELISA data (not shown). Whereas, in LH mutant mice, a change in the mobility of adiponectin monomers on SDS-PAGE (Fig. 3C) and a reduction of the total adiponectin level (data not shown) were observed. These data also confirm the ELISA results. Adiponectin from the LH mutant serum migrated faster than the wild type, and the apparent size difference varied from 130 Da to 1400 Da, which corresponds to a loss of one to four Glc-Gal-Hyl residues, calculated using the theoretical molecular mass of 340 Da for Glc-Gal-Hyl. These results are supported by our mass spectrometry data of adiponectin produced in LH mutant MEFs and clearly indicate that the loss of the lysyl hydroxylase activity of LH3 in LH mutant mice also affects the modification of lysine residues in adiponectin *in vivo*.

### The secretion of adiponectin is disturbed in LH mutant mice

Adiponectin is secreted into the blood stream mainly by adipocytes [30]. The substitution of the four conserved lysine residues in the collagenous domain of adiponectin with arginines has been shown to retard the secretion rate, which suggests that the attachment of glycosyl and hydroxyl groups to lysine residues is important for the secretion of adiponectin [18], [19]. In order to evaluate whether the reduced serum concentration of adiponectin in LH mutant mice is due to disturbed secretion, we analyzed the total adiponectin level in adipose tissue of LH mutant mice. ELISA analysis indicated no significant changes in the adiponectin levels in the adipose tissue of the LH mutant when compared with the wild type (data not shown). In 10 months old LH mutant male mice the reduction was 8% and in females 18%, whereas in 5 month old males no decrease was detected. This corresponds well with the immunoblot analyses where no dramatic changes in the adiponectin level of adipose tissue were seen (Fig. 3D). These results indicated no profound change in the production of adiponectin in the LH mutant adipose tissues, which was further supported by the unchanged mRNA level of adiponectin in the adipose tissue of LH mutant mice (data not shown). However, the immunoblot analysis of adipose tissue indicated, as in serum, a reduction in the molecular weight of the adiponectin monomer in LH mutant adipose tissue (Fig. 3D). As expected based on the serum results, no difference in the amount or in the mobility of adiponectin was detected on immunoblots of adiponectin from adipose tissue of LH3^+/−^ mice compared with wild type (Fig. 3E). Our results suggest that without the lysyl hydroxylase activity of LH3 the adiponectin produced is insufficiently modified in the adipose tissue of LH mutant mice and thus is not efficiently secreted into the circulation. Normal leptin levels in LH mutant mice (Fig. 3B) suggest that the reduction of adiponectin is not due to a general defect in secretion. Our *in vitro* and *in vivo* results confirm that lysine modifications catalyzed by LH3 are important for the secretion of adiponectin and even a partial loss of lysine modifications disturbs the secretion *in vivo*.

### The distribution of oligomeric forms of adiponectin is altered in LH mutant mice

**Figure 4:**
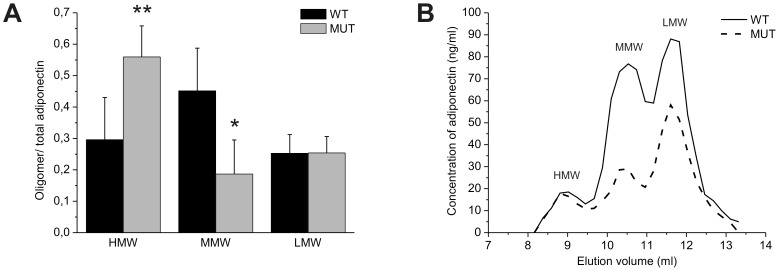
The distribution of adiponectin oligomers is altered in the serum of LH mutant mice. (A) The ratio of HMW/total adiponectin was significantly increased and MMW/total decreased in serum of 2 months old male mice, even though the total amount of adiponectin was lowered as seen in the elution profile of adiponectin (B). The oligomeric forms were separated with gel filtration chromatography and quantified either from adiponectin immunoblots (A) or by ELISA (B). The level of oligomeric forms (A) was calculated as a proportion of total adiponectin. The values represent the average ± SD of the experiments. P values were calculated using unpaired homoscedastic student t-tests with two-tailed distribution.* p<0.05, ** p<0.01, *** p<0.001.

In order to analyze how the changes in LH3 activities affect the assembly of adiponectin oligomers, the distribution of oligomers in sera of LH3^+/−^ and LH mutant mice was analyzed by fractionating adiponectin complexes using gel filtration chromatography. In 5 months old LH3^+/−^ male and female mice no significant changes were observed in the oligomer distribution compared with wild type mice (not shown). In LH mutant mice fractionation of the oligomeric adiponectin forms showed a significant increase in the HMW/total adiponectin ratio and a decrease in the MMW/total adiponectin ratio when compared with the wild type (Fig. 4A and 4B). However, after correcting for the reduction of total adiponectin in serum seen in Fig. 3A, the calculated HMW level in the LH mutant serum was on average 77%±5 of the wild type, and the MMW and LMW levels were on average 36%±6 and 48%±21 of the wild type, respectively. The reduction of the total adiponectin level in LH mutant mice can also been seen in the elution profile from gel filtration chromatography (Fig. 4B), where especially the concentration of adiponectin in fractions corresponding to MMW and LMW forms is reduced. Our data suggest that the partial loss of lysine modifications affect the formation of adiponectin oligomers leading to substantially lowered secretion levels of all adiponectin forms when taking into an account of the decreased total adiponectin level.

### The metabolic regulation is affected in the LH mutant mice

Adiponectin is an adipokine controlling the glucose and lipid metabolism of the body [16], [31]. In order to determine whether the inactivation of LH activity of LH3 and reduced level and altered oligomer distribution of adiponectin in the LH mutant mice affect the metabolic regulation *in vivo*, 4 months old female LH mutant and wild type mice were kept for four months on a high fat diet (60% kcal from fat). The high fat diet was used since in adiponectin knockout mouse studies the effect of decreased adiponectin level on glucose and lipid metabolism was diet-inducible [32], [33]. After the high fat diet, the average body weight of the LH mutants was significantly higher than that of the wild type mice (Table 2). In addition, adiponectin levels were reduced by 40% in the LH mutant mice when compared with the wild type (p 0,034). The HMW amount in LH mutant serum was 85% of the wild type level and the MMW and LMW levels were on average 38% and 63% of the wild type MMW and LMW amount, respectively (data not shown). The distribution of adiponectin oligomers is not affected by the high fat diet when compared with the results of mice on a standard chow. However, LDL, triglyceride and the blood glucose levels were significantly higher in the LH mutant mice when compared with the wild type (Table 2). Moreover, the insulin level was increased by 40% in the LH mutant serum in comparison with the wild type, although the difference was statistically only marginally significant (p 0.063) due to the great variation in values (Table 2). Interestingly, the male LH mutant mice, when fed the 42% kcal/fat diet, did not show significant metabolic changes compared with the wild type mice, although they did have altered adiponectin levels (Table S1). This implies that there might be other, possibly sex-dependent factors that also affect the phenotype of the female and male LH mutant mice fed with high fat diet.

**Table 2:** Comparison of serum parameters of 8 months old female LH mutant and wild type mice after four months of high fat diet.

	Wild type	LH mutant	p○
Weight*	40.5±6.2	43.8±4.4	*
Lipase∧	46.7±8.1	47.8±16.5	ns
Cholesterol#	3.23±0.49	3.25±0.53	ns
HDL#	1.45±0.15	1.44±0.16	ns
LDL#	0.17±0.04	0.22±0.08	**
Triglycerides#	0.50±0.11	0.61±0.13	**
FFA#	1.14±0.12	1.16±0.11	ns
Glucose#	9.97±1.77	11.5±1.83	**
Insulin¤	3.1±1.1	4.4±2.3	ns
Adiponectin¤	17502±2048	10775±1226	***

* weight (g);

∧ lipase (U/l);

# cholesterol, HDL, LDL, triglycerides, FFA and glucose (mmol/l);

¤ insulin and adiponectin (ng/ml) ± S.D. Wild type n = 18, LH mutant n = 25, except for insulin measurements wild type n = 13 and LH mutant n = 15.

○ P values were calculated using unpaired homoscedastic student t-test with two-tailed distribution.

* p<0.05,

** p<0.01,

*** p<0.001.

Abbreviations: HDL = High-density lipoprotein, LDL = Low-density lipoprotein, FFA = FreeFatty Acids.

**Figure 5:**
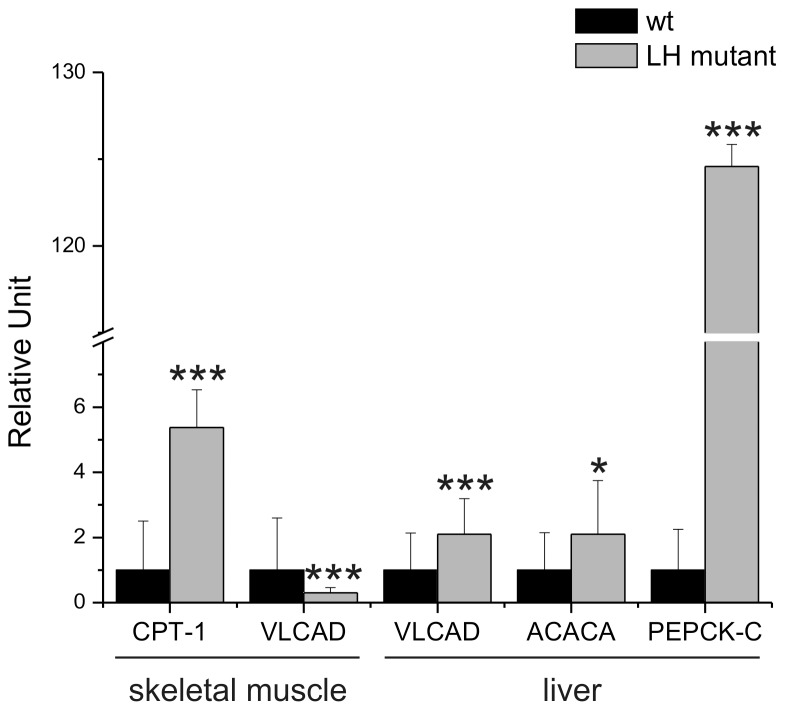
The expression of genes involved in β-oxidation, fatty acid synthesis and gluconeogenesis in LH mutant mice. Quantitative RT-PCR was used to determine changes in the gene expressions in LH mutant mice after 4 months of high fat diet. The columns represent the relative expression levels of muscular genes involved in the mitochondrial β-oxidation; CPT-1 and VLCAD and hepatic genes involved in the mitochondrial β-oxidation, fatty acid synthesis and gluconeogenesis; VLCAD, ACACA and PEPCK-C, respectively. For relative quantification of gene expression, the results were normalized using GADPH and β-actin as endogenous controls, and the expression of the wild type samples were set to 1. Results represent means with 95% confidence interval of 10 independently analyzed mice for each genotype. P values were calculated using unpaired heteroscedastic student t-test with one-tailed distribution. * p<0.05, ** p<0.01, *** p<0.001. Abbreviations: CPT-1 = carnitine palmitoyltransferase, VLCAD = very long chain acyl-CoA dehydrogenase, Acaca = acetyl-CoA carboxylase, PEPCK-C = phosphoenoylpyruvate carboxykinase.

We also investigated whether the high fat diet changes the gene expression of enzymes involved in mitochondrial fatty acid oxidation and gluconeogenic pathway in LH mutant mice. Adiponectin is known to stimulate fatty acid oxidation and suppress hepatic glucose production [34]. When the expression of mitochondrial β-oxidation enzymes in skeletal muscle were compared between wild type and LH mutant mice after the high fat diet, a 5-fold increase was detected in the expression levels of CPT-1, the rate-limiting enzyme of β-oxidation, in LH mutant mice (Fig. 5). The expression level of another mitochondrial β-oxidation enzyme, VLCAD, was decreased by 5-fold in LH mutant skeletal muscle (Fig. 5), but in liver it was increased by 2-fold. The expression of Acaca, which catalyzes the first step in the fatty acid synthesis pathway, was also increased by 2-fold in the liver of the LH mutant. The expression of one of the key enzymes of gluconeogenesis, PEPCK-C, was dramatically increased by 124-fold in LH mutant liver (Fig. 5). Our quantitative PCR data together with blood parameters suggest that the metabolic regulation is affected in the LH mutant mice as seen as changes in the lipid and glucose metabolism.

## Discussion

We have shown earlier that the loss of multifunctional LH3 has dramatic consequences in collagens due to the lack of Glc-Gal-Hyl structures [8], [9]. In addition to collagens there are a number of other proteins that contain a short collagenous domain in their structure, and many of these proteins have lysine residues in the correct position to be modified. Adiponectin is one of these proteins where the presence and function of hydroxylated and glycosylated lysine residues have been indicated [17]–[19]. Before this study it was not known which enzyme catalyzes the formation of these specific lysine modifications in adiponectin.

In this report we show that recombinant adiponectin produced in the total absence of LH3 lacked glucosylation of Glc-Gal-Hyl modifications known to exist in the collagenous domain and which are important for the insulin-sensitizing actions adiponectin. Our mass spectrometry analyses indicated that lysines of adiponectin are modified only to Gal-Hyl residues in the absence of LH3 and indicate that LH3 is the only enzyme capable to further glucosylate Gal-Hyl residues. This is supported by results, which show that the suppression of LH3 decreases the extend of glucosylation of Gal-Hyl residues in type I collagen [35]. Our results suggest that LH1 and LH2, the other two isoforms in lysyl hydroxylase family, can most probably compensate the loss of the lysyl hydroxylase activity of LH3 during the adiponectin biosynthesis at least when no LH3 protein is present in the cells. This also confirms our earlier results showing that lysyl hydroxylase isoforms do not have absolute substrate specificity [36], and normal levels of LH1 and LH2 cannot fully compensate the LH activity of LH3 [9]. Collagen β(1-*O*)galactosyltransferases, GLT25D1 and GLT25D2, have been recently reported to catalyze the galactosylation of hydroxylysine residues in type I–V collagens and mannan-binding lectin [37]. Based on our results it seems possible that that GLT25D1 and GLT25D2 or some other galactosyltransferase can also galactosylate hydroxylysine residues of adiponectin. Our results further indicate that especially the Glc units of Glc-Gal-Hyl residues are crucial for formation of HMW and MMW adiponectin and thus affect the secretion of adiponectin. These data emphasize the significance of LH3 and especially its glucosyltransferase activity in biosynthesis of adiponectin.

When adiponectin was produced in the LH mutant cells in which the lysyl hydroxylase activity of LH3 has been inactivated by an Asp669Ala point mutation our analyses indicated more variation in the amount of lysine hydroxylation and glycosylation when compared with the wild type cells. In addition adiponectin analyzed from the serum of LH mutant mice showed the reduction in the molecular weight confirming a partial loss of the Glc-Gal-Hyl residues. These data show that LH activity of LH3 is needed to complete modification of adiponectin lysine residues. Furthermore, the amount of circulating adiponectin was significantly reduced due to the disturbance of the secretion of adiponectin from adipose tissue to blood circulation in the LH mutant serum. As shown elsewhere, when the four conserved lysine residues were mutated to arginines and adiponectin expressed in HEK293 cells, the HMW forms were hardly detectable, whereas substitution of any single lysine residue caused only a slight reduction in the formation of the HMW adiponectin [19]. Our earlier results from collagens show that the lack of lysyl hydroxylase activity of LH3 in LH mutant mice did not affect tetramerization of type VI collagen. However, the tetramers of type VI collagen formed massive aggregates inside cells and in the extracellular matrix of LH mutant mice [9]. The bulky Glc-Gal-Hyl residues are thought to decrease the electrostatic and hydrophobic interactions between collagen fibrils and thereby decrease interfibrillar interactions [38]. Therefore it is possible that the increased HMW/total adiponectin ratio detected in the serum of LH mutant mice might be a consequence of the abnormal aggregation of partially hydroxylated and glycosylated LMW and MMW oligomers.

The decreased total adiponectin and HMW levels in our LH mutant mice suggested possible defects in glucose metabolism and fatty acid oxidation [16], [39], [40], since decreased serum levels of adiponectin and especially the decrease of the HMW isoform are known to correlate with metabolic syndrome traits [16], [21], [31]. In heterozygous adiponectin knockout mice already a 40–70% reduction in plasma adiponectin was associated with insulin resistance [41]. Significant increases of triglyceride, LDL and glucose levels and weight suggest a development of metabolic syndrome in LH mutant mice fed a high fat diet. The diet-induced changes in our LH mutant mice resemble those seen in total and heterozygous adiponectin knockout mice [14], [32], [41], [42] suggesting that there might be an association between reduced adiponectin level and changes observed. In addition to the blood parameter, the changes in the expression of genes involved in the mitochondrial fatty acid metabolism and gluconeogenesis further suggest that the metabolic regulation is affected due to deficiency of the LH activity of LH3. In addition to adiponectin there are other collagenous adipokines, such as C1q/TNF-related proteins (CTRP1-9) [43]–[45], which might be affected due to impaired lysine modifications and thus influence the development of metabolic phenotype in our LH mutant mice.

## Conclusions

In this study we have shown for the first time that LH3 catalyzes the posttranslational lysine modifications of adiponectin and especially the glucosylation activity of LH3 is critical. The total absence of LH3 fully prevented the formation of HMW and MMW oligomers which resulted from the lack of Glc units in Gal-Hyl residues of adiponectin. Our results indicate for the first time that the Glc unit of Glc-Gal-Hyl residue is the most critical modification for the formation of high molecular weight oligomers of adiponectin and even loss of some of the Glc-Gal-Hyl residues is enough to reduce the level of adiponectin in our LH mutant mice. Changes in the glucose, triglyceride and LDL levels in LH mutant mice reflect that the metabolic regulation is affected in these mice. The indication that LH3 regulates the posttranslational lysine modification of adiponectin makes it a potential candidate for the development of future therapeutic treatments for various metabolic and cardiovascular abnormalities associated with obesity and insulin resistance, which are linked to the dysregulation of adiponectin secretion.

## Supporting Information

Figure S1 **MALDI TOF mass spectra of tryptic peptide mixtures.** The purified recombinant adiponectin produced in wild type, LH3^−/−^ knockout and LH mutant MEFs were in gel digested with trypsin and the peptide mixtures were analyzed with MALDI TOF MS. Analysis of adiponectin produced in LH3^−/−^ knockout MEFs revealed a set of fragments with unique masses compared with wild type recombinant adiponectin. Adiponectin produced in LH mutant MEFs was mainly trypsinized to fragments with similar masses as found in wild type. Abbreviations: WT = wild type; KO = LH3^−/−^ knockout; MUT = LH mutant.
(TIF)

Table S1 Comparison of serum parameters of male LH mutant and wild type mice after three months of a high fat diet.
(DOCX)

## References

[1] Heikkinen J, Risteli M, Wang C, Latvala J, Rossi M, et al (2000) Lysyl hydroxylase 3 is a multifunctional protein possessing collagen glucosyltransferase activity. J Biol Chem 275 (46) 36158–36163. 10934207 10.1074/jbc.M006203200

[2] Wang C, Luosujärvi H, Heikkinen J, Risteli M, Uitto L, et al (2002) The third activity for lysyl hydroxylase 3: Galactosylation of hydroxylysyl residues in collagens in vitro. Matrix Biol 21 (7) 559–566. 12475640 10.1016/s0945-053x(02)00071-9

[3] Myllylä R, Wang C, Heikkinen J, Juffer A, Lampela O, et al (2007) Expanding the lysyl hydroxylase toolbox: New insights into the localization and activities of lysyl hydroxylase 3 (LH3). J Cell Physiol 212 (2) 323–329. 17516569 10.1002/jcp.21036

[4] Gelse K, Pöschl E, Aigner T (2003) Collagens—structure, function, and biosynthesis. Adv Drug Deliv Rev (55) 1531. 14623400 10.1016/j.addr.2003.08.002

[5] Salo AM, Wang C, Sipilä L, Sormunen R, Vapola M, et al (2006) Lysyl hydroxylase 3 (LH3) modifies proteins in the extracellular space, a novel mechanism for matrix remodeling. J Cell Physiol 207 (3) 644–653. 16447251 10.1002/jcp.20596

[6] Salo AM, Sipilä L, Sormunen R, Ruotsalainen H, Vainio S, et al (2006) The lysyl hydroxylase isoforms are widely expressed during mouse embryogenesis, but obtain tissue- and cell-specific patterns in the adult. Matrix Biol 25 (8) 475–483. 16996725 10.1016/j.matbio.2006.08.260

[7] Wang C, Kovanen V, Raudasoja P, Eskelinen S, Pospiech H, et al (2009) The glycosyltransferase activities of lysyl hydroxylase 3 (LH3) in the extracellular space are important for cell growth and viability. J Cell Mol Med 13 (3) 508–521. 18298658 10.1111/j.1582-4934.2008.00286.x PMC3822511

[8] Ruotsalainen H, Sipilä L, Vapola M, Sormunen R, Salo AM, et al (2006) Glycosylation catalyzed by lysyl hydroxylase 3 is essential for basement membranes. J Cell Sci 119 (Pt 4) 625–635. 16467571 10.1242/jcs.02780

[9] Sipilä L, Ruotsalainen H, Sormunen R, Baker NL, Lamande SR, et al (2007) Secretion and assembly of type IV and VI collagens depend on glycosylation of hydroxylysines. J Biol Chem 282 (46) 33381–33388. 17873278 10.1074/jbc.M704198200

[10] Salo AM, Cox H, Farndon P, Moss C, Grindulis H, et al (2008) A connective tissue disorder caused by mutations of the lysyl hydroxylase 3 gene. Am J Hum Genet 83 (4) 495–503. 18834968 10.1016/j.ajhg.2008.09.004 PMC2561927

[11] Risteli M, Ruotsalainen H, Salo AM, Sormunen R, Sipilä L, et al (2009) Reduction of lysyl hydroxylase 3 causes deleterious changes in the deposition and organization of extracellular matrix. J Biol Chem 284 (41) 28204–28211. 19696018 10.1074/jbc.M109.038190 PMC2788872

[12] Tilg H, Moschen AR (2006) Adipocytokines: Mediators linking adipose tissue, inflammation and immunity. Nat Rev Immunol 6 (10) 772–783. 16998510 10.1038/nri1937

[13] Yun JE, Sull JW, Lee HY, Park E, Kim S, et al (2009) Serum adiponectin as a useful marker for metabolic syndrome in type 2 diabetic patients. Diabetes Metab Res Rev 25 (3) 259–265. 19214966 10.1002/dmrr.946

[14] Kangas-Kontio T, Huotari A, Ruotsalainen H, Herzig KH, Tamminen M, et al (2010) Genetic and environmental determinants of total and high-molecular weight adiponectin in families with low HDL-cholesterol and early onset coronary heart disease. Atherosclerosis 210 (2) 479–485. 20056223 10.1016/j.atherosclerosis.2009.12.022

[15] Vlasova M, Purhonen AK, Järvelin MR, Rodilla E, Pascual J, et al (2010) Role of adipokines in obesity-associated hypertension. Acta Physiol (Oxf) 200 (2) 107–127. 20653609 10.1111/j.1748-1716.2010.02171.x

[16] Wang Y, Lam KS, Yau MH, Xu A (2008) Post-translational modifications of adiponectin: Mechanisms and functional implications. Biochem J 409 (3) 623–633. 18177270 10.1042/BJ20071492

[17] Wang Y, Xu A, Knight C, Xu LY, Cooper GJ (2002) Hydroxylation and glycosylation of the four conserved lysine residues in the collagenous domain of adiponectin. potential role in the modulation of its insulin-sensitizing activity. J Biol Chem 277 (22) 19521–19529. 11912203 10.1074/jbc.M200601200

[18] Richards AA, Stephens T, Charlton HK, Jones A, Macdonald GA, et al (2006) Adiponectin multimerization is dependent on conserved lysines in the collagenous domain: Evidence for regulation of multimerization by alterations in posttranslational modifications. Mol Endocrinol 20 (7) 1673–1687. 16497731 10.1210/me.2005-0390

[19] Wang Y, Lam KS, Chan L, Chan KW, Lam JB, et al (2006) Post-translational modifications of the four conserved lysine residues within the collagenous domain of adiponectin are required for the formation of its high molecular weight oligomeric complex. J Biol Chem 281 (24) 16391–16400. 16621799 10.1074/jbc.M513907200

[20] Waki H, Yamauchi T, Kamon J, Ito Y, Uchida S, et al (2003) Impaired multimerization of human adiponectin mutants associated with diabetes. molecular structure and multimer formation of adiponectin. J Biol Chem 278 (41) 40352–40363. 12878598 10.1074/jbc.M300365200

[21] Pajvani UB, Hawkins M, Combs TP, Rajala MW, Doebber T, et al (2004) Complex distribution, not absolute amount of adiponectin, correlates with thiazolidinedione-mediated improvement in insulin sensitivity. J Biol Chem 279 (13) 12152–12162. 14699128 10.1074/jbc.M311113200

[22] Schraw T, Wang ZV, Halberg N, Hawkins M, Scherer PE (2008) Plasma adiponectin complexes have distinct biochemical characteristics. Endocrinology 149 (5) 2270–2282. 18202126 10.1210/en.2007-1561 PMC2329278

[23] Wang C, Ristiluoma MM, Salo AM, Eskelinen S, Myllylä R (2011) Lysyl hydroxylase 3 is secreted from cells by two pathways. J Cell Physiol 227 (2) 668–75. 10.1002/jcp.22774 21465473

[24] Xu A, Chan KW, Hoo RL, Wang Y, Tan KC, et al (2005) Testosterone selectively reduces the high molecular weight form of adiponectin by inhibiting its secretion from adipocytes. J Biol Chem 280 (18) 18073–18080. 15760892 10.1074/jbc.M414231200

[25] Miinalainen IJ, Schmitz W, Huotari A, Autio KJ, Soininen R, et al (2009) Mitochondrial 2,4-dienoyl-CoA reductase deficiency in mice results in severe hypoglycemia with stress intolerance and unimpaired ketogenesis. PLoS Genet 5 (7) e1000543. 19578400 10.1371/journal.pgen.1000543 PMC2697383

[26] Wu GY, Pereyra B, Seifter S (1981) Specificity of trypsin and carboxypeptidase B for hydroxylysine residues in denatured collagens. Biochemistry 20 (15) 4321–4324. 7284327 10.1021/bi00518a013

[27] Combs TP, Berg AH, Rajala MW, Klebanov S, Iyengar P, et al (2003) Sexual differentiation, pregnancy, calorie restriction, and aging affect the adipocyte-specific secretory protein adiponectin. Diabetes 52 (2) 268–276. 12540596 10.2337/diabetes.52.2.268

[28] Nishizawa H, Shimomura I, Kishida K, Maeda N, Kuriyama H, et al (2002) Androgens decrease plasma adiponectin, an insulin-sensitizing adipocyte-derived protein. Diabetes 51 (9) 2734–2741. 12196466 10.2337/diabetes.51.9.2734

[29] Plaisance EP, Grandjean PW, Judd RL, Jones KW, Taylor JK (2009) The influence of sex, body composition, and nonesterified fatty acids on serum adipokine concentrations. Metabolism 58 (11) 1557–1563. 19592049 10.1016/j.metabol.2009.04.038 PMC7134378

[30] Scherer PE, Williams S, Fogliano M, Baldini G, Lodish HF (1995) A novel serum protein similar to C1q, produced exclusively in adipocytes. J Biol Chem 270 (45) 26746–26749. 7592907 10.1074/jbc.270.45.26746

[31] Lara-Castro C, Fu Y, Chung BH, Garvey WT (2007) Adiponectin and the metabolic syndrome: Mechanisms mediating risk for metabolic and cardiovascular disease. Curr Opin Lipidol 18 (3) 263–270. 17495599 10.1097/MOL.0b013e32814a645f

[32] Maeda N, Shimomura I, Kishida K, Nishizawa H, Matsuda M, et al (2002) Diet-induced insulin resistance in mice lacking adiponectin/ACRP30. Nat Med 8 (7) 731–737. 12068289 10.1038/nm724

[33] Nawrocki AR, Rajala MW, Tomas E, Pajvani UB, Saha AK, et al (2006) Mice lacking adiponectin show decreased hepatic insulin sensitivity and reduced responsiveness to peroxisome proliferator-activated receptor gamma agonists. J Biol Chem 281 (5) 2654–2660. 16326714 10.1074/jbc.M505311200

[34] Liu M, Liu F (2010) Transcriptional and post-translational regulation of adiponectin. Biochem J 425 (1) 41–52. 10.1042/BJ20091045 20001961

[35] Sricholpech M, Perdivara I, Nagaoka H, Yokoyama M, Tomer KB, et al (2011) Lysyl hydroxylase 3 glucosylates galactosylhydroxylysine residues in type I collagen in osteoblast culture. J Biol Chem 286 (11) 8846–8856. 21220425 10.1074/jbc.M110.178509 PMC3058983

[36] Risteli M, Niemitalo O, Lankinen H, Juffer AH, Myllyla R (2004) Characterization of collagenous peptides bound to lysyl hydroxylase isoforms. J Biol Chem 279 (36) 37535–37543. 15208310 10.1074/jbc.M405638200

[37] Schegg B, Hulsmeier AJ, Rutschmann C, Maag C, Hennet T (2009) Core glycosylation of collagen is initiated by two beta(1-O)galactosyltransferases. Mol Cell Biol 29 (4) 943–952. 19075007 10.1128/MCB.02085-07 PMC2643808

[38] Mizuno K, Adachi E, Imamura Y, Katsumata O, Hayashi T (2001) The fibril structure of type V collagen triple-helical domain. Micron 32 (3) 317–323. 11006511 10.1016/s0968-4328(00)00036-6

[39] Kadowaki T, Yamauchi T, Kubota N, Hara K, Ueki K, et al (2006) Adiponectin and adiponectin receptors in insulin resistance, diabetes, and the metabolic syndrome. J Clin Invest 116 (7) 1784–1792. 16823476 10.1172/JCI29126 PMC1483172

[40] Sheng T, Yang K (2008) Adiponectin and its association with insulin resistance and type 2 diabetes. J Genet Genomics 35 (6) 321–326. 18571119 10.1016/S1673-8527(08)60047-8

[41] Kubota N, Terauchi Y, Yamauchi T, Kubota T, Moroi M, et al (2002) Disruption of adiponectin causes insulin resistance and neointimal formation. J Biol Chem 277 (29) 25863–25866. 12032136 10.1074/jbc.C200251200

[42] Qiao L, Zou C, van der Westhuyzen DR, Shao J (2008) Adiponectin reduces plasma triglyceride by increasing VLDL triglyceride catabolism. Diabetes 57 (7) 1824–1833. 18375436 10.2337/db07-0435 PMC2453618

[43] Wong GW, Krawczyk SA, Kitidis-Mitrokostas C, Ge G, Spooner E, et al (2009) Identification and characterization of CTRP9, a novel secreted glycoprotein, from adipose tissue that reduces serum glucose in mice and forms heterotrimers with adiponectin. FASEB J 23 (1) 241–258. 18787108 10.1096/fj.08-114991 PMC2626616

[44] Wong GW, Wang J, Hug C, Tsao TS, Lodish HF (2004) A family of Acrp30/adiponectin structural and functional paralogs. Proc Natl Acad Sci U S A 101 (28) 10302–10307. 15231994 10.1073/pnas.0403760101 PMC478567

[45] Wong GW, Krawczyk SA, Kitidis-Mitrokostas C, Revett T, Gimeno R, et al (2008) Molecular, biochemical and functional characterizations of C1q/TNF family members: Adipose-tissue-selective expression patterns, regulation by PPAR-gamma agonist, cysteine-mediated oligomerizations, combinatorial associations and metabolic functions. Biochem J 416 (2) 161–177. 18783346 10.1042/BJ20081240 PMC3936483

