# Supplementary material for: The Activities of Lysyl Hydroxylase 3 (LH3) Regulate the Amount and Oligomerization Status of Adiponectin
Source: PLoS One. 2012 Nov 29;7(11):e50045. doi: 10.1371/journal.pone.0050045 (PMC3510199; doi:10.1371/journal.pone.0050045)
Supplement: Table S1 — Comparison of serum parameters of male LH mutant and wild type mice after three months of a high fat diet. (DOCX) [file pone.0050045.s002.docx]

Table S1 Comparison of serum parameters of male LH mutant and wild type mice after three months of a high fat diet.

|  | wt | LH mut | p^¤^ |
| --- | --- | --- | --- |
| Weight ^#^ | 35.7±4.5 | 33.5±3.2 | ns |
| Lipase ^#^ | 55.5±20.3 | 39.0±8.0 | * |
| Cholesterol ^#^ | 3.3±1.0 | 2.9±0.4 | ns |
| HDL-C ^#^ | 1.5±0.4 | 1.5±0.2 | ns |
| LDL-C ^#^ | 0.2±0.1 | 0.2±0.03 | ns |
| Triglycerides^#^ | 0.59±0.13 | 0.68±0.13 | ns |
| FFA ^#^ | 1.0±0.3 | 1.1±0.2 | ns |
| Glucose ^#^ | 12.8±2.2 | 13.7±3.6 | ns |
| Adiponectin ^#^ | 7469±2713 | 3265±784 | *** |

^#^ weight (g), lipase (U/l); cholesterol, HDL-C, LDL- C, triglycerides, FFA and glucose (mmol/l), adiponectin (ng/ml) ± S.D. n= 10 for each group.

^¤^ P values were calculated using unpaired homoscedastic student t-test with two-tailed distribution.

* p < 0.05, ** p < 0.01, *** p < 0.001

Abbreviations: HDL = High-density lipoprotein, LDL = Low-density lipoprotein, FFA = .Free Fatty Acids.
